# High light intensity enhances cannabinoid biosynthesis through concerted gene expression in hemp (*Cannabis sativa*) flowers

**DOI:** 10.3389/fpls.2025.1687794

**Published:** 2025-10-21

**Authors:** Seungyong Hahm, Gwonjeong Bok, Sungjin Kim, Byungjun Kim, Yongjae Lee, Sunwoo Kim, Jongseok Park

**Affiliations:** ^1^ Department of Horticultural Science, Chungnam National University, Daejeon, Republic of Korea; ^2^ Glocal University Project Team, Sunchon National University, Suncheon, Jeonnam, Republic of Korea; ^3^ Department of Bio-AI Convergence, Chungnam National University, Daejeon, Republic of Korea

**Keywords:** cannabinoids, industrial cannabis, inflorescence, photosynthesis, plant factory

## Abstract

**Introduction:**

Research on optimizing light intensity to maximize phytochemical production during hemp flowering is limited, despite growing global demand. We investigated the effects of light-emitting diode (LED) intensity on hemp growth, cannabinoid content, and gene expression.

**Methods:**

Hemp plants (*Cannabis sativa* 'Queen Dream') were grown under white LEDs at light intensities of 200, 400, and 600 μmol·m^−2^·s^−1^ with a 12/12 h photoperiod for 35 days during the flowering stage.

**Results:**

The dry mass of stems, leaves, and flowers increased linearly with increasing light intensity. Cannabinoid analysis revealed that levels of cannabidiol (CBD), cannabidiolic acid, and tetrahydrocannabinolic acid increased linearly with light intensity, reaching the highest levels at 600 μmol·m^−2^·s^−1^. Total CBD increased by 36.88% at 600 μmol·m^−2^·s^−1^ compared to 200 μmol·m^−2^·s^−1^. CBD yield per plant also increased linearly across the entire light intensity range. Gene expression analysis revealed a coordinated upregulation of genes involved in the hexanoate–olivetolic acid–cannabigerolic acid–cannabinoid biosynthesis pathway under high light intensity, with a notable increase in cannabidiolic acid synthase (*CBDAS*) expression.

**Conclusion:**

These findings demonstrate that a light intensity of 600 μmol·m^−2^·s^−1^ effectively enhances both biomass and cannabinoid accumulation at the flowering stage, providing valuable insights for controlled-environment hemp cultivation aimed at maximizing CBD yield.

## 1 Introduction

Hemp *(Cannabis sativa)* belongs to the Cannabaceae family (Small, 1976). *C*. *sativa* contains cannabinoids such as Δ9-tetrahydrocannabinol (THC) and cannabidiol (CBD) (ElSohly et al., 2017). It is commonly known as marijuana or hemp, where hemp is defined as *C. sativa* containing less than 0.3% Δ9-THC (Tagen and Klumpers, 2022). Hemp is used for fiber, seed, and cannabinoid extraction, with cannabinoids primarily concentrated in the flowers (Crispim Massuela et al., 2022). The therapeutic properties of cannabinoids, particularly CBD, are widely acknowledged in healthcare (Nelson et al., 2020). Cannabinoids are effective in treating medical conditions by interacting with brain receptors, particularly in the management of epilepsy and pain (Kow et al., 2014; Mostafavi and Gaitanis, 2020). They have the potential to treat thrombosis, atopic disease, and insomnia (Wilkinson and Williamson, 2007; Ahmed et al., 2015; Aso and Ferrer, 2016; Pisanti et al., 2017; Vigil et al., 2018; Hacke et al., 2019). These therapeutic properties have driven global efforts toward legalization. The global demand for hemp is increasing, with an annual production growth rate of 26.21% (Kolodinsky and Lacasse, 2021; Teleszko et al., 2022).

The increasing market demand has accelerated the expansion of controlled environmental hemp production. Vertical farming systems that use artificial lighting provide multiple benefits, including better transplant quality and more efficient resource utilization (Kozai, 2013; Tian et al., 2021). Research on various crops, especially leafy greens, has consistently demonstrated that vertical farming systems can achieve improved yields and product quality compared with conventional methods (Zhang et al., 2018; Sabatino et al., 2019; Chowdhury et al., 2021). In vertical farming systems, hemp can achieve six harvest cycles annually, which greatly enhances profitability through efficient vertical space utilization (UNODC, 2009). Artificial lighting in vertical setups enables precise adjustment of yield and quality by managing light intensity. Other studies on drug-type *C*. *sativa* have suggested that increased light intensity supports growth and floral biomass production within specific intensity ranges (Llewellyn et al., 2022; Rodriguez-Morrison et al., 2021). However, excessive light intensity can reduce photosynthetic efficiency and plant productivity, requiring careful light management (Nelson et al., 2010; Tombesi et al., 2015; Cho et al., 2019). Light management becomes particularly critical during the flowering stage of hemp, when phytochemicals are produced (Eichhorn Bilodeau et al., 2019). The underlying mechanism involves light-activating photoreceptors that trigger changes in gene expression, ultimately promoting phytochemical biosynthesis (Mawphlang and Kharshiing, 2017).

Light intensity modulates phytochemical accumulation in a species-specific manner, with elevated intensities increasing cannabinoids in hemp and phenolic compounds in vegetables (Colonna et al., 2016; Pérez-López et al., 2018). A complex enzymatic pathway centered on cannabigerolic acid (CBGA) enables the synthesis of cannabinoids. The precursor compounds for CBGA formation are olivetolic acid (OA), which is synthesized by olivetolic acid synthase (OLS), olivetolic acid cyclase (OAC), and geranyl pyrophosphate (GPP), which is produced by geranyl pyrophosphate synthase (GPPS) (Taura et al., 2009; Luo et al., 2019). This key intermediate results from the enzymatic reaction of OA with GPP facilitated by geranyl pyrophosphate-olivetolate geranyl transferase (PT) (Fellermeier et al., 2001). The different cannabinoids found in hemp are produced through the enzymatic conversion of CBGA by three stereoselective enzymes, each of which synthesizes a unique acidic cannabinoid. Tetrahydrocannabinolic acid synthase (THCAS) creates THCA, cannabidiolic acid synthase (CBDAS) forms cannabidiolic acid (CBDA), and cannabichromenic acid synthase (CBCAS) produces CBCA through specific cyclization processes (Tahir et al., 2021). Understanding and controlling this enzymatic pathway is crucial for optimizing cultivation practices, as environmental factors such as light can influence the gene expression of these enzymes, directly affecting cannabinoid production in hemp flowers.

Despite the growing global demand for medical hemp, research on maximizing phytochemical yield through light intensity management during flowering is limited, particularly for controlled-environment agriculture systems. Although the general effects of light on cannabinoid biosynthesis have been studied, specific protocols for vertical farming systems, where precise environmental control enables year-round production, require further investigation. This study aimed to determine the optimal light intensity for maximizing growth and phytochemical accumulation during hemp flowering in vertical farms, and to evaluate the transcriptional responses of cannabinoid biosynthesis genes under different light intensities. This research addresses a critical knowledge gap in the rapidly expanding controlled-environment hemp industry.

## 2 Materials and methods

### 2.1 Plant materials and cutting conditions

Hemp (*Cannabis sativa* ‘Queen Dream’) stock plants (mother plants) were maintained under a 20/4 (day/night) h photoperiod with a mean canopy-level light intensity of 300 (± 10) μmol·m^−2^·s^−1^ using high-pressure sodium (HPS) lamps (250 W, E39; Il-Kwang Co., Seoul, Korea). Relative humidity and temperature were set at 70 (± 10)% and 25°C, respectively, in a cultivation room. Plants were grown for four months in a cultivation room, and cuttings were taken when stem length exceeded 2 m. Hemp cuttings (10 cm long) with three fully expanded leaves were collected from stock plants. Each cutting was rooted in rockwool growing substrate (Grodan AX Plug; Grodan Inc., Roermond, Netherlands) (25 × 25 × 40 mm) and arranged in trays at a density of 200 plants·m^−2^. The cutting trays were then moved to a growing chamber with the relative humidity and air temperature set at 90% and 25°C, respectively. The hemp cuttings were irrigated daily with tap water. Adventitious roots appeared two weeks after cutting. Uniform cuttings were then transplanted into cultivation beds. After rooting, uniform cuttings were transferred to an experimental growth system.

### 2.2 Growth conditions

**Figure 1:**
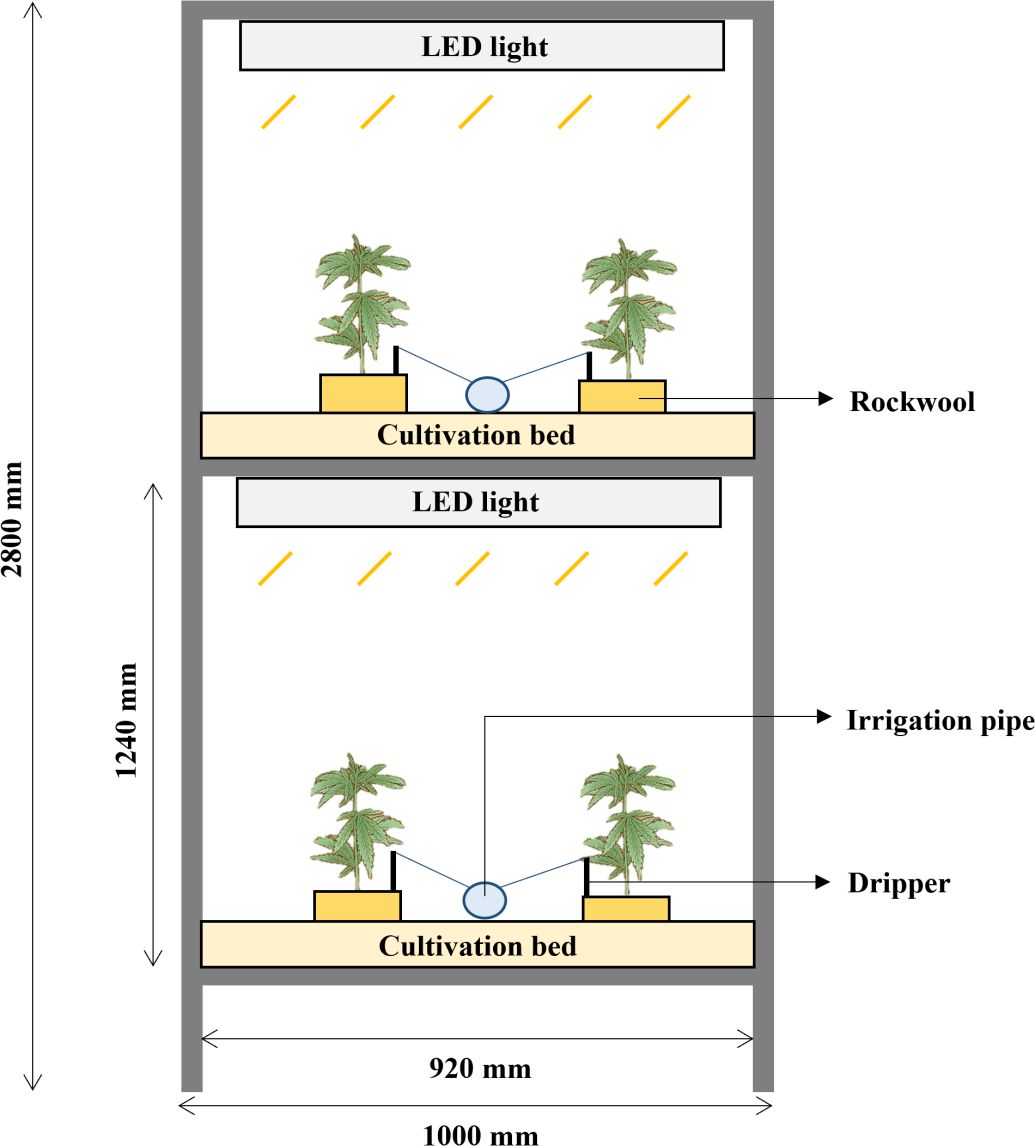
Experimental setup of the two-layer vertical cultivation system. The system features LED lighting for each layer, cultivation bed with rockwool substrates, and an automated irrigation system with drippers for nutrient supply. Dimensions: 1000 mm (W) × 2800 mm (H), with each layer measuring 920 mm (W) × 1240 mm (H).

The cultivation bed frame was made of an aluminum profile (40 mm × 40 mm). The cultivation room contained four two-layer vertical cultivation systems. Each module measured 1000 mm (width) × 2000 mm (length) × 2800 mm (height) (**Figure 1**). The distance between the light source at each layer and the bottom of the cultivation bed was 1240 mm, and a nutrient solution tank (2000 L) was placed beside the cultivation beds. A submersible pump (PD-G050M; WILO Pumps Ltd., Busan, Korea) was installed in the nutrient solution tank to supply nutrient solution to the cultivation beds at each stage, with one dripper per plant. After planting, the electrical conductivity (EC) of the Hoagland nutrient solution was maintained at 2.0 ± 0.2 dS·m^−1^, pH 6.2 ± 0.1, and the EC and pH were measured once every two days. The cultivation room (5000 mm [length] × 3500 mm [width] × 3000 mm [height]), designed as an enclosed artificial light plant factory, was maintained at 22 ± 2/20 ± 2°C (day/night), with 50–70% relative humidity using a heat pump (TH/MMU-AP0244HP-K; Toshiba Carrier Co. Ltd., Seoul, Korea), chiller (Unit Cooler BSU-030E; SUNGJIN Co., Ltd., Seoul, Korea), and humidifier (HU-4200C; Ohsungsa Co., Ltd., Seoul, Korea) to control the temperature and humidity.

### 2.3 Light treatment

**Figure 2:**
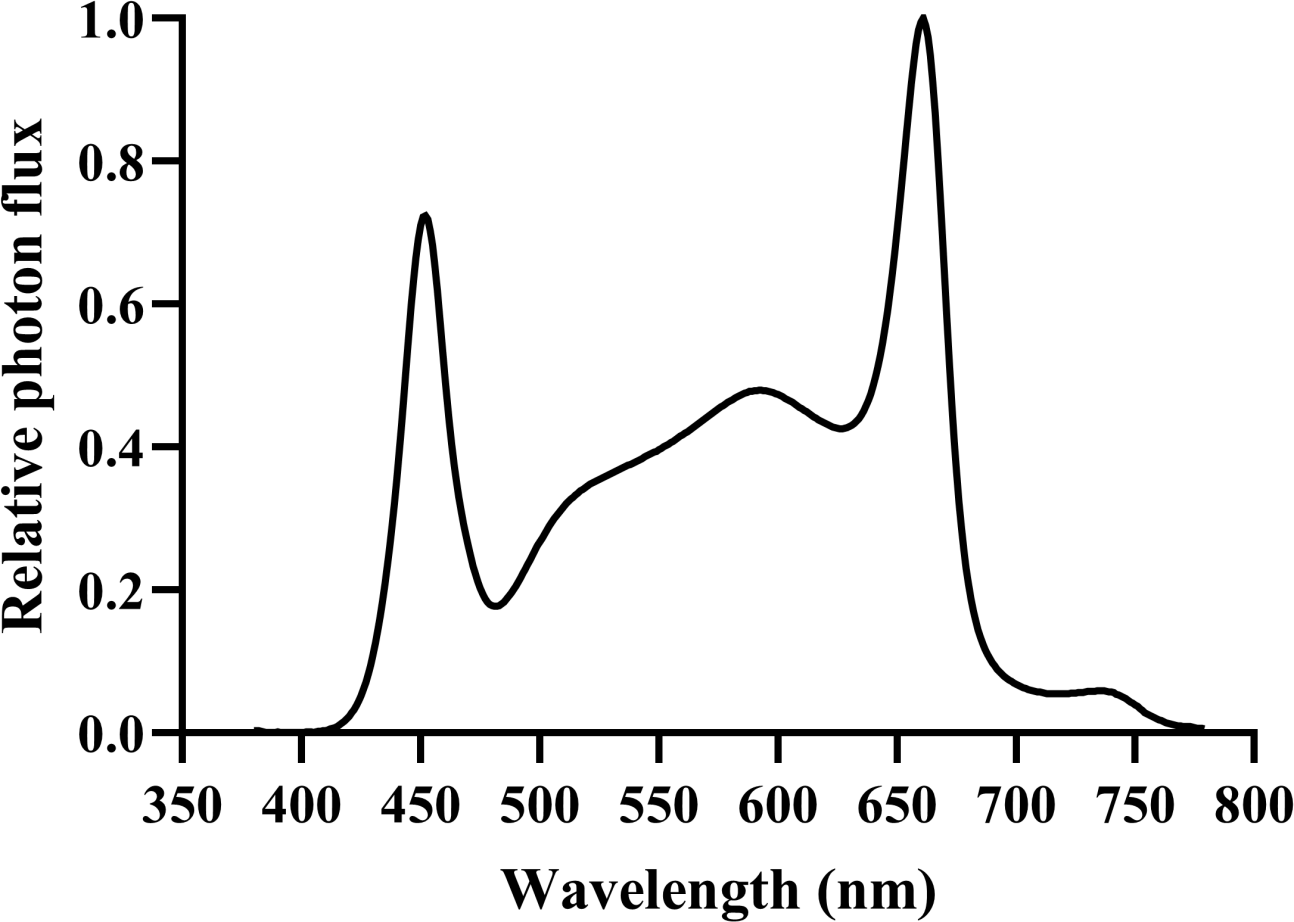
Spectral distribution of the white LED lighting system used in the experiment. The relative photon flux is normalized to a maximum value of 1.0. Measurements were taken using a portable spectroradiometer (LI-180) in the 380–780 nm range at 1 nm intervals.

The light-emitting diode (LED) light source (APACK Inc., Daejeon, Korea) and light spectrum distribution used in this study were measured with a portable spectroradiometer (LI-180; LI-COR Inc., Lincoln, NE, USA) in the 380–780 nm range at 1 nm intervals (**Figure 2**). The photosynthetic photon flux density (μmol·m^−2^·s^−1^) of each light-treatment group was measured using a photon sensor (LI-190; LI-COR Inc., Lincoln, NE, USA). The light intensity was measured at the bottom surface of the cultivation beds. Vegetative growth was maintained at 400 ± 10 μmol·m^−2^·s^−1^ and a 20/4 h photoperiod using dimming with the light intensity control device for 28 days. During the 35-day of flowering stage, hemp plants were cultivated at 200, 400, and 600 μmol·m^−2^·s^−1^ with a 12/12 h photoperiod. The experiment was conducted three times with all three light treatments tested during each experiment, using one plant per treatment (*n* = 3).

### 2.4 Measurement of plant growth parameters

Plant growth was measured following the flowering stage, with individual plants treated as experimental replicates for four different growth parameters (*n* = 3). Plant height was measured using a tape measure (KMC-220; Komelon Co., Ltd., Busan, Korea). All plant parts were harvested from each plant without differentiation by position, immediately frozen in liquid nitrogen, and stored in a deep freezer (−80°C; ULT-669; GMS Co. Ltd., Dongducheon, Korea) until analysis. After samples were dried in a freeze dryer (TFD8508, IlshinBioBase Co., Ltd., Dongducheon, Korea) at −85°C for seven days, the dry mass (stems, leaves and flowers) was measured using an ultrafine scale (CAS MWII-300H; CAS Co., Ltd., East Rutherford, NJ, USA; precision: 0.01 g). Stems and leaves were analyzed for dry mass measurement but not for cannabinoid content, as flowers contain the highest cannabinoid concentrations and represent the primary commercial product.

### 2.5 The cannabinoid assay and CBD yield

The cannabinoid assay was performed according to a modified protocol adapted from Hahm et al. (2025). Dried flowers (100 mg) were extracted in 2 mL methanol/hexane solvent mixture (9:1, v/v) by sonication at ambient temperature (25 °C) for 20 min, followed by centrifugation at 13,000 rpm for 5 min. The resulting extract (1 mL) was filtered through a 0.45 μm syringe filter prior to the analysis. High-performance liquid chromatography (HPLC) analysis employed an Agilent 1260 system (Agilent Technologies, Inc., Santa Clara, CA, USA) equipped with a Poroshell 120 EC-C18 analytical column (4.6 mm × 50 mm, 2.7 μm; Agilent Technologies, Inc., Santa Clara, CA, USA). Chromatographic conditions included: UV detection at 210 nm, mobile phase flow rate of 1 mL/min, and column temperature maintained at 25 °C. Compounds were separated by gradient elution with mobile phases A (0.1% formic acid in water) and B (0.1% formic acid in acetonitrile). The gradient program spanned a total runtime of 35 min: initial mobile phase B concentration at 55% for 5 min, linear increase to 85% B over 20 min (5–25 min), isocratic hold at 85% B for 5 min (25–30 min), rapid return to 55% B (30–30.1 min), and re-equilibration at 55% B for 35 min. Reference standards for CBDA (CAS No. CBD-1735) and cannabidiol (CAS No. THC-303), and THCA (CAS No. THC-741) were purchased from Lipomed (Arleisheim, Switzerland). Calibration curves were constructed using six concentration points with the following linear equations: CBDA (y = 32.251x + 19.131), CBD (y = 83.907x − 24.125), and THCA (y = 31.180x + 35.423). The calibration range extended from 50 to 1,000 µg·mL^−1^ for all analytes. Total CBD concentration was determined using the formula: Total CBD = CBDA (mg·g^−1^ DW) × 0.877 + CBD (mg·g^−1^ DW), incorporating the molecular weight conversion factor for CBDA decarboxylation. Additionally, CBD yield per plant was calculated by multiplying flower dry mass (g/plant) by total CBD concentration (mg·g^−1^ DW).

### 2.6 Identification of cannabinoid biosynthesis genes

Cannabinoid biosynthesis gene sequences for *C*. *sativa* were retrieved from published literature through the GenBank (https://www.ncbi.nlm.nih.gov/genbank/). Supplementary sequence information was obtained from the Cannabis GDB database (https://gdb.supercann.net/). Complete cDNA sequences for seven target genes (*THCAS*, *CBCAS*, *CBDAS*, *GPPS*, *OLS*, *OAC*, and *PT*) were selected from *C*. *sativa* L. genomic resources, corresponding to GenBank accession numbers CsCAN_00G0198470, CsPK_00G0121830, CsCAN_00G0257910, CsCAN_00G0073620, CsJLD_00G0306020, CsLAC_00G0215960, and CsCBD_01G0018110, respectively. Primer development was performed using the Primer Quest online tool (https://www.idtdna.com/pages/tools/primerquest) to generate gene-specific amplification primers, yielding amplicon products ranging from 90 to 100 bp in length (**Supplementary Table 1**).

### 2.7 Total RNA isolation, cDNA synthesis, and quantitative real-time polymerase chain reaction

Hemp flowers were collected for RNA extraction, followed by reverse transcription PCR (RT-PCR) and quantitative RT-PCR (qRT-PCR). Flowers were collected from 35-day flowering stage plants under three distinct light intensity conditions. Primers specific to the target genes were developed using the predicted sequences within the designated regions. Each sample contained three biological replicates, which were promptly flash-frozen in liquid nitrogen and preserved at −80°C in a deep freezer (ULT-669; GMS Co. Ltd., Dongducheon, Korea). RNA was isolated from 100 mg of tissue sample by grinding with a mortar and pestle under liquid nitrogen conditions, followed by the addition of 1 mL of TRIzol reagent (5 Prime; Gaithersburg, MD, USA). First-strand cDNA synthesis for qRT-PCR was accomplished using the ReverTra Ace-α-kit (Toyobo, Osaka, Japan) with oligo (dT) 20 primers from the extracted total RNA. Expression analysis of the seven cannabinoid biosynthesis-associated genes was conducted by quantitative real-time PCR using a Mini Opticon Real-time PCR system (Bio-Rad Laboratories, Hercules, CA, USA). The thermal cycling protocol included: initial denaturation at 95°C for 3 min; followed by 35 amplification cycles consisting of 95°C for 15 s, 54°C for 20 s, and 72°C for 15 s; with final steps of 95°C for 10 s and 65°C for 5 s. PCR reactions were performed in 20 μL volumes containing 0.4 μM primer concentrations and 1× SYBR Green Real-Time PCR master mix (Toyobo, Osaka, Japan). Relative gene expression quantification was determined through the 2^−ΔΔCt^ calculation method.

### 2.8 Statistical analysis

Statistical analyses were performed using SPSS (version 29.0.2.0; SPSS Inc., Chicago, IL, USA). Individual plants were treated as experimental replicates for statistical analysis (*n* = 3). Data were tested for normality using the Shapiro-Wilk test and homogeneity of variance using Levene’s test. One-way analysis of variance (ANOVA) followed by Tukey’s multiple range test was performed to determine significant differences among treatment means at *P* < 0.01. Linear regression analysis was conducted to evaluate the dose-response relationships between the light intensity treatments and the measured parameters. Each treatment included three biological replicates with three technical replicates per biological replicate for gene expression analysis. All figures were created using GraphPad Prism (version 10.5; GraphPad Software, Boston, Massachusetts, USA).

## 3 Results

### 3.1 Growth parameters

**Figure 3:**
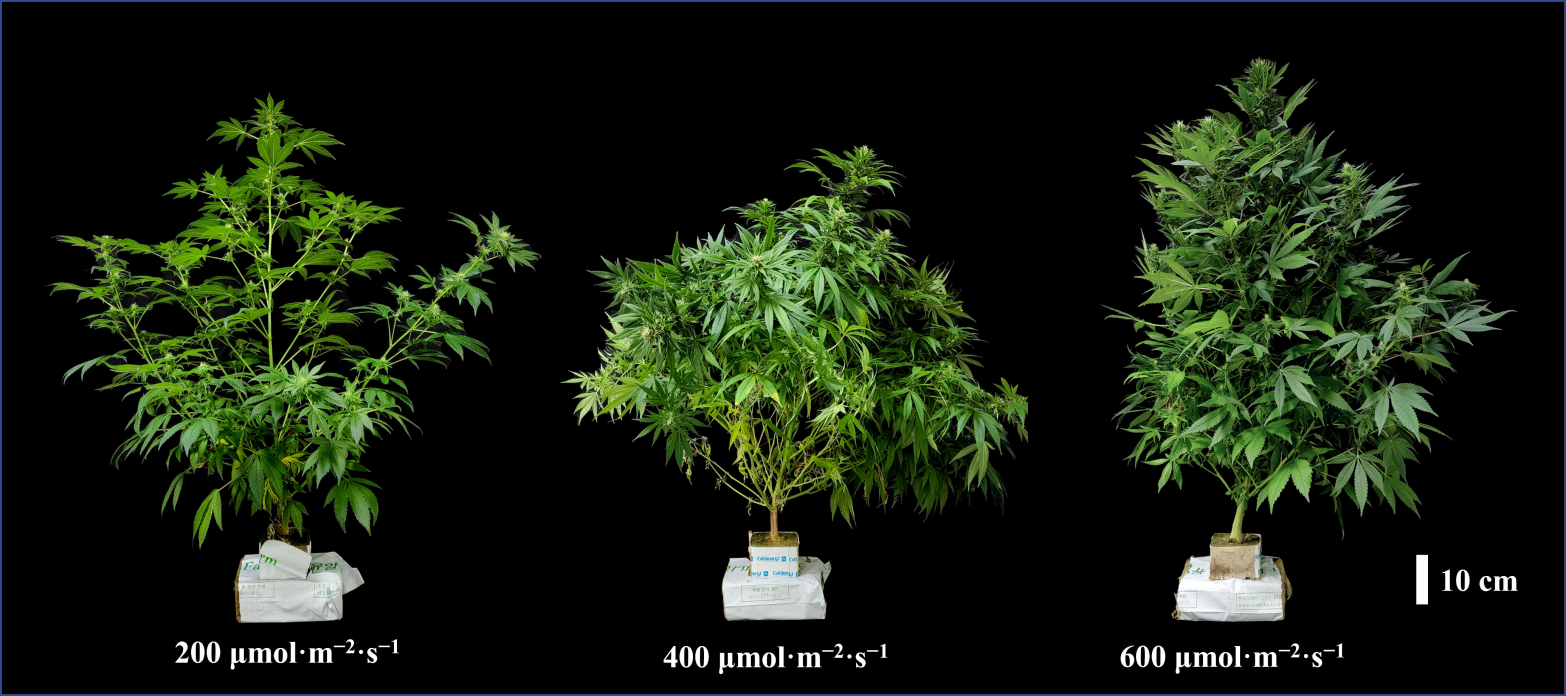
Morphological differences in hemp (*Cannabis sativa* ‘Queen Dream’) plants grown under different light intensities for 35 days during flowering stage. Plants were cultivated under different light intensities (200, 400, and 600 μmol·m^−2^·s^−1^). Scale bar = 10 cm.

**Figure 4:**
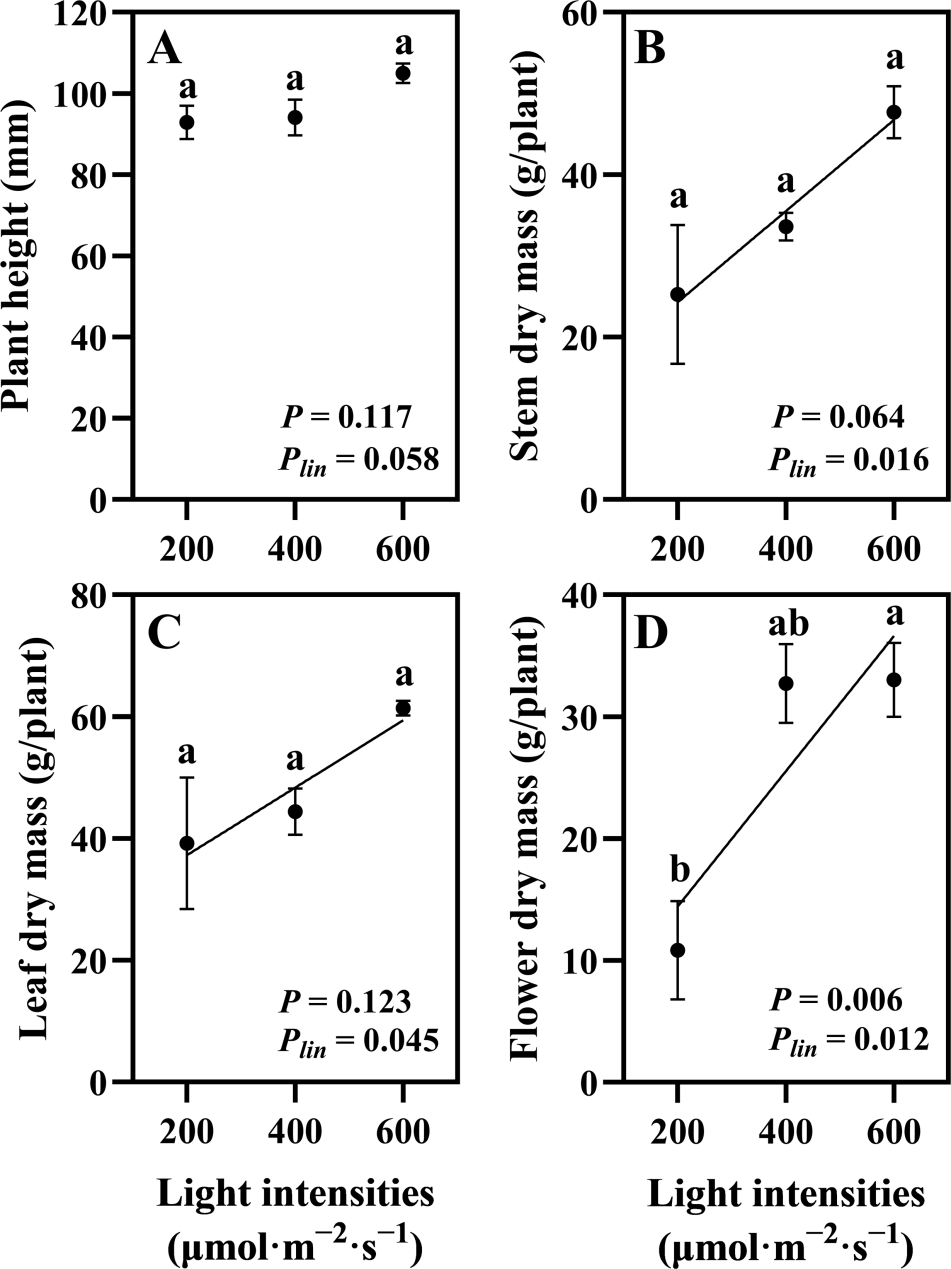
Effects of light intensity on hemp (*Cannabis sativa* ‘Queen Dream’) growth parameters. **(A)** Plant height, **(B)** stem dry mass, **(C)** leaf dry mass, and **(D)** flower dry mass of hemp plants grown under three different light intensities (200, 400, and 600 μmol·m^−2^·s^−1^) for 35 days during flowering stage. Data were analyzed using one-way ANOVA followed by Tukey’s test. *P_lin_* indicates the significance of linear trend analysis across light intensity treatments. Different letters indicate significant differences between treatments (*P* < 0.01). Data are presented as mean ± standard error (*n* = 3).

Uniform plants were selected after 28 days of the vegetative stage and subsequently cultivated for 35 days under flowering with three different light intensity treatments (200, 400, and 600 μmol·m^−2^·s^−1^). The hemp plants exhibited distinct morphological responses to different light intensities. Plants grown under 200 μmol·m^−2^·s^−1^ showed notably wider internodal spacing, indicating a shade avoidance response (**Figure 3**). Plant height showed no linear relationship with light intensity (*P_lin_* = 0.058; **Figure 4A**). Stem dry mass (*P* = 0.064, *P_lin_* = 0.016) and leaf dry mass (*P* = 0.123, *P_lin_* = 0.045) showed no significant differences among different light intensities, but a linear increasing trend was observed (**Figures 4B, C**). Flower dry mass increased by 205% at 600 compared to 200 μmol·m^−2^·s^−1^ and showed a linear increase (*P* = 0.006, *P_lin_* = 0.012) (**Figure 4D**). These findings indicate continuous increase in shoot dry mass in hemp plants up to 600 μmol·m^−2^·s^−1^.

### 3.2 Cannabinoid content and CBD yield

**Figure 5:**
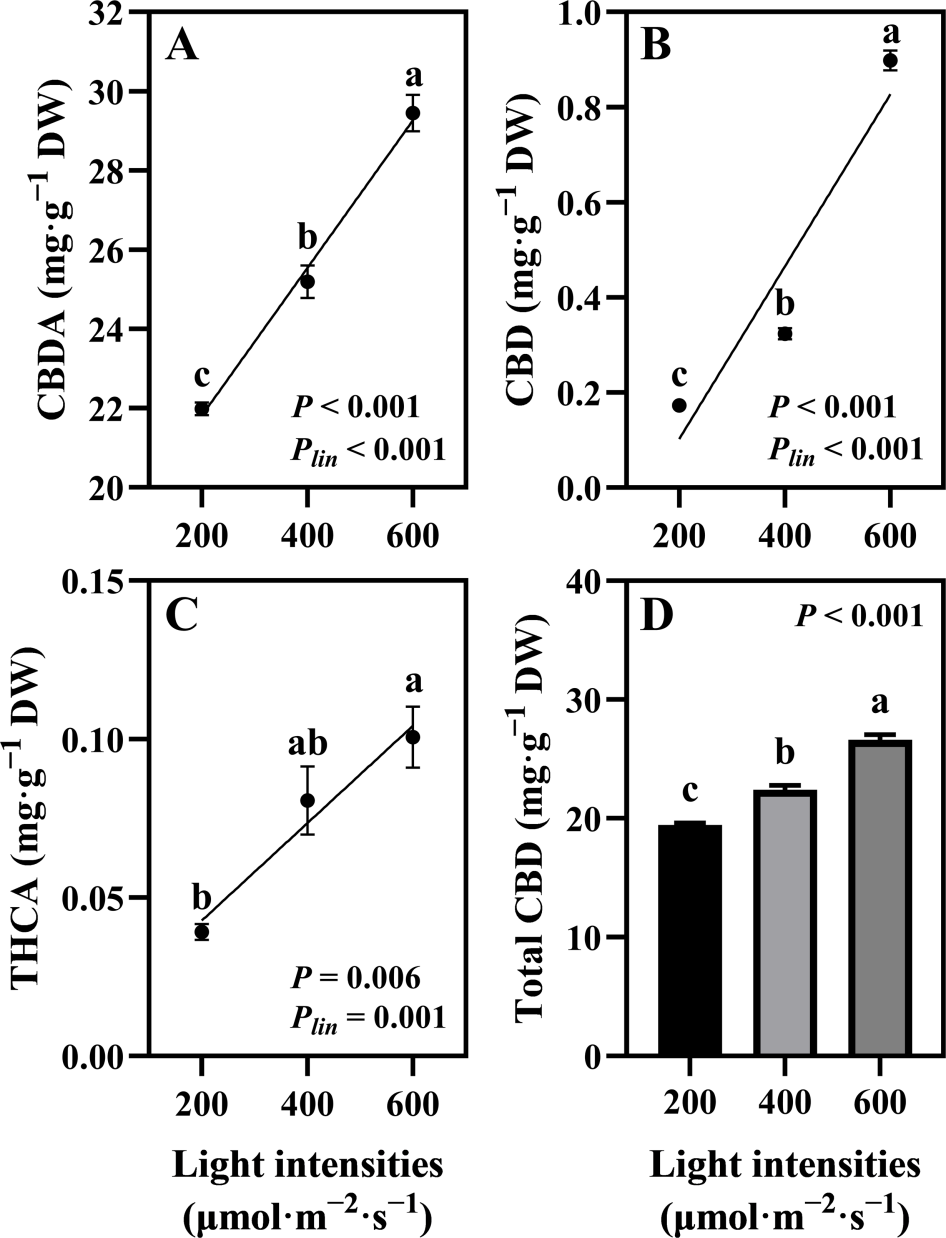
Effects of light intensity on cannabinoid content in hemp (*Cannabis sativa* ‘Queen Dream’) flowers. **(A)** cannabidiolic-acid (CBDA) content, **(B)** cannabidiol (CBD) content, **(C)** tetrahydrocannabinolic acid (THCA) content, and **(D)** Total CBD content in hemp flowers grown under three different light intensities (200, 400, and 600 μmol·m^−2^·s^−1^) for 35 days during flowering stage. Data were analyzed using one-way ANOVA followed by Tukey’s test. *P_lin_* indicates the significance of linear trend analysis across light intensity treatments. Different letters indicate significant differences between treatments (*P* < 0.01). Data are presented as mean ± standard error (*n* = 3).

**Figure 6:**
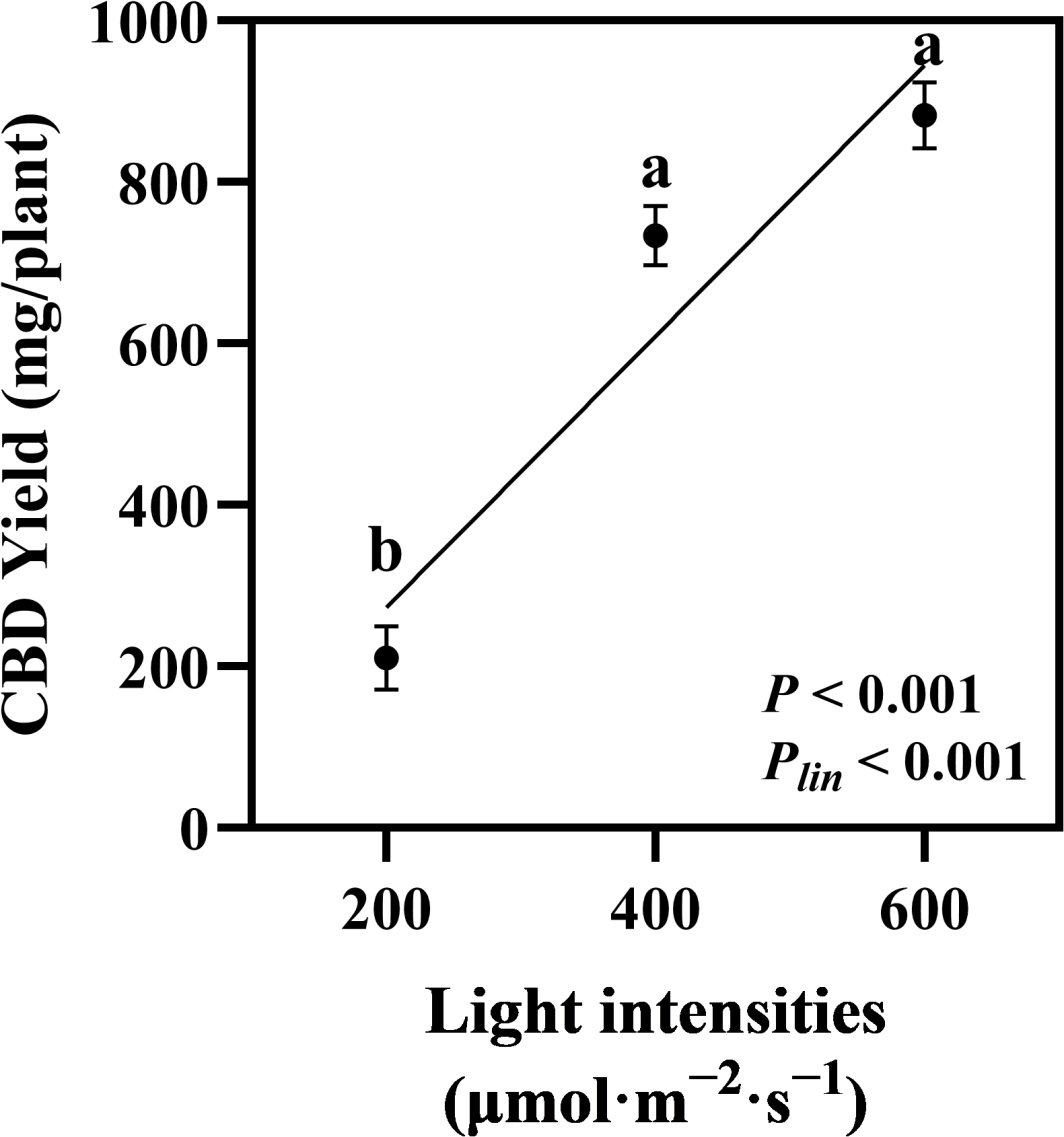
Effects of light intensity on CBD yield in hemp (*Cannabis sativa* ‘Queen Dream’). CBD yield per plant of hemp plants grown under three different light intensities (200, 400, and 600 μmol·m^−2^·s^−1^) for 35 days during flowering stage. Data were analyzed using one-way ANOVA followed by Tukey’s test. *P_lin_* indicates the significance of linear trend analysis across light intensity treatments. Different letters indicate significant differences between treatments (*P* < 0.01). Data are presented as mean ± standard error (*n* = 3).

The quantified cannabinoids, CBDA, CBD, and THCA, showed the highest accumulation in hemp during the flowering stage under 600 μmol·m^−2^·s^−1^ light intensity, with linear accumulation confirmed across the 200–600 μmol·m^−2^·s^−1^ range (*P_lin_* < 0.001, < 0.001, and = 0.001, respectively; **Figures 5A–C**). Total CBD showed a significant increase of 36.88% in hemp grown under 600 μmol·m^−2^·s^−1^ compared with that under the 200 μmol·m^−2^·s^−1^ treatment (**Figure 5D**). CBD yield per plant increased significantly by 248% when light intensity increased from 200 to 400 μmol·m^−2^·s^−1^. When light intensity increased from 400 to 600 μmol·m^−2^·s^−1^, CBD yield showed a 20.31% increase, though this difference was not statistically significant. Linear increases in CBD yield were confirmed across the 200–600 μmol·m^−2^·s^−1^ light intensity range (*P_lin_* < 0.001; **Figure 6**).

### 3.3 Relative gene expression of cannabinoid biosynthesis enzyme

**Figure 7:**
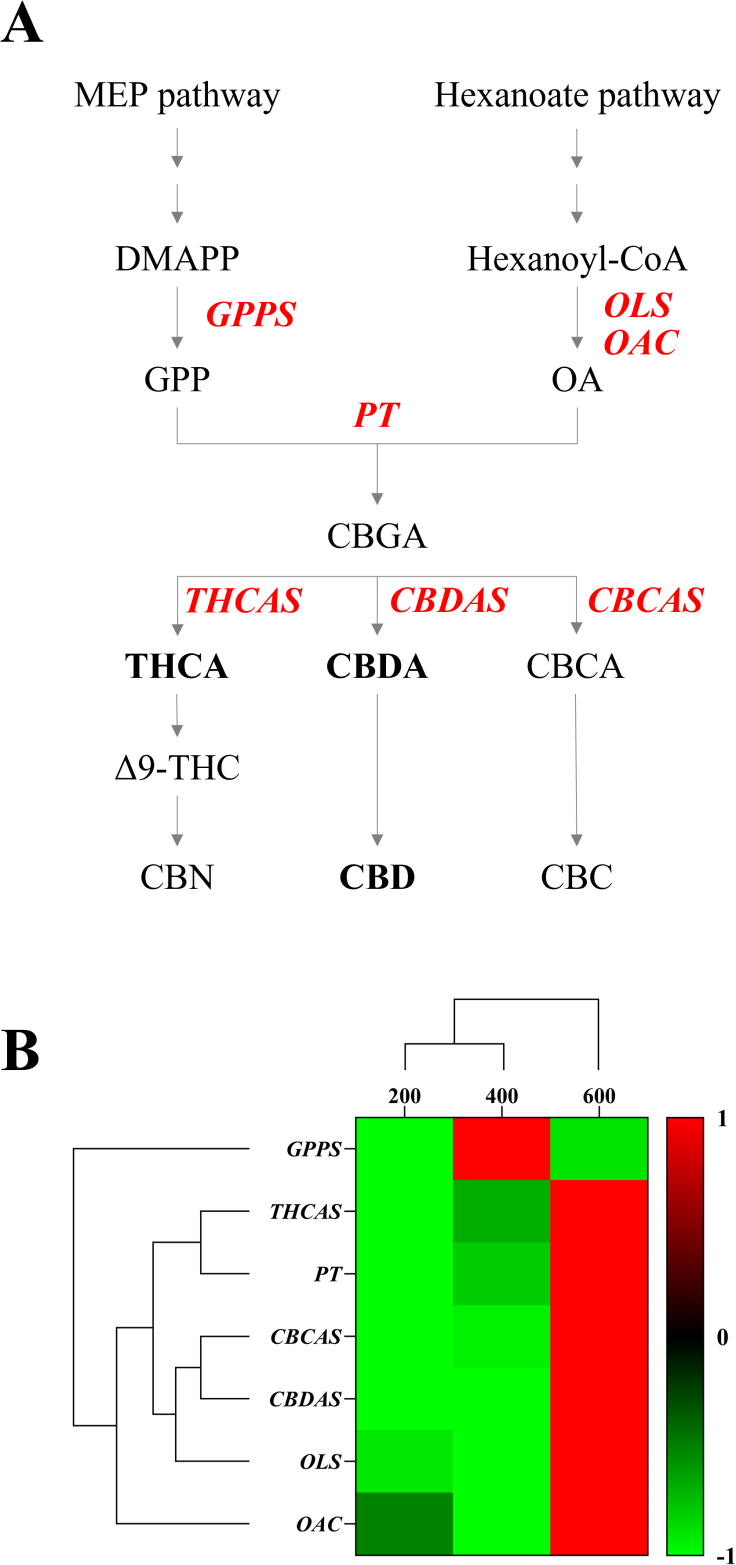
Cannabinoid biosynthesis pathway and gene expression analysis under different light intensities (200, 400, and 600 μmol·m^−2^·s^−1^). **(A)** Schematic representation of the cannabinoid biosynthesis pathway showing the MEP pathway leading to geranyl pyrophosphate (GPP) production and the hexanoate pathway leading to olivetolic acid (OA) formation. Key enzymes are highlighted in red: *GPPS* (geranyl pyrophosphate synthase), *OLS* (olivetolic acid synthase), *OAC* (olivetolic acid cyclase), *PT* (geranyl pyrophosphate-olivetolate geranyl transferase), and cannabinoid synthases (tetrahydrocannabinolic acid synthase, *THCAS*; cannabichromenic acid synthase, *CBCAS*; cannabidiolic-acid synthase, *CBDAS*). **(B)** Hierarchical clustering and heatmap analysis of relative gene expression levels under three light intensities (200, 400, and 600 μmol·m^−2^·s^−1^). The color scale represents normalized expression levels from -1 (green, low expression) to +1 (red, high expression).

**Figure 8:**
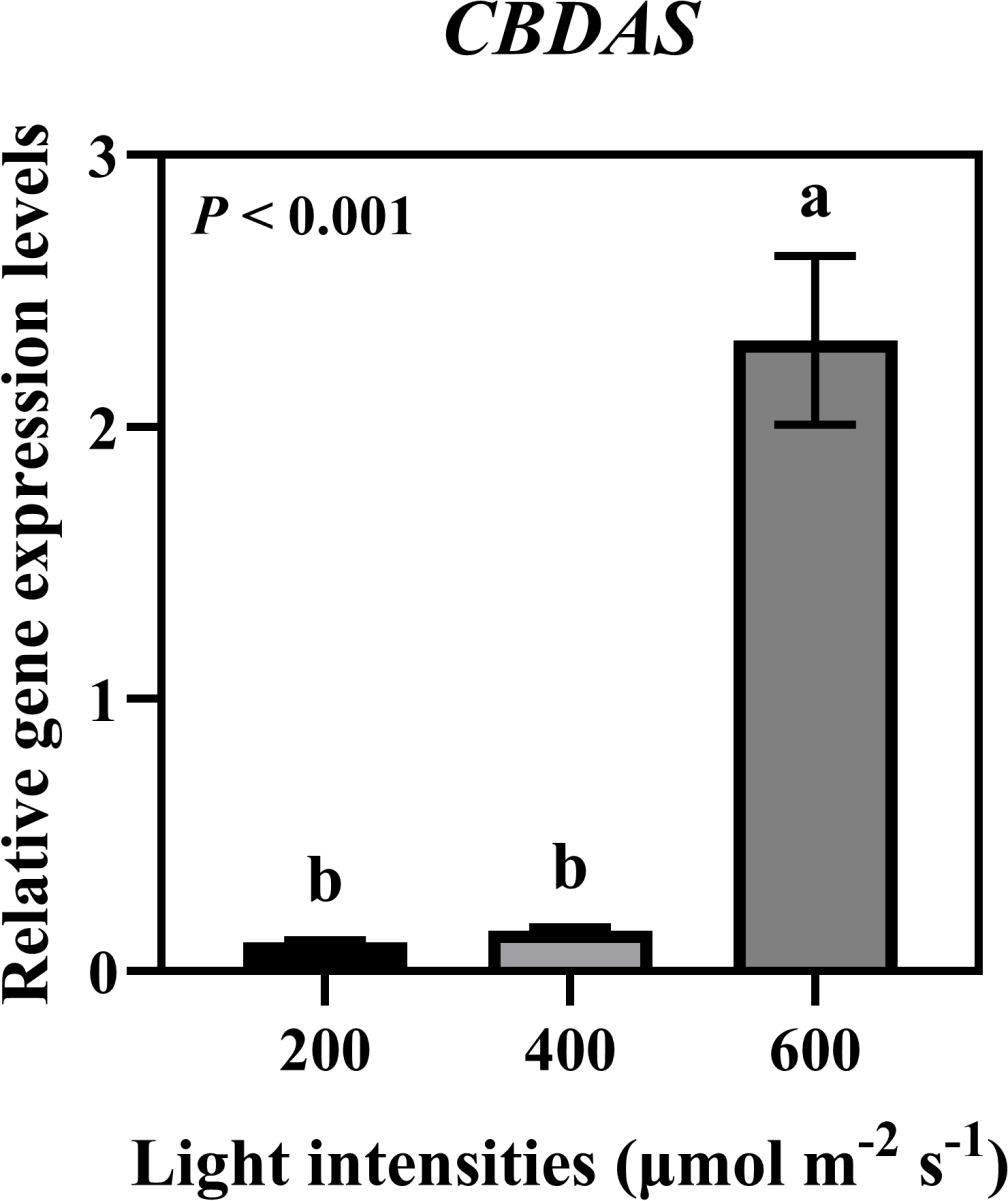
Relative gene expression levels of cannabidiolic acid synthase (*CBDAS*) under different light intensities (200, 400, and 600 μmol·m^−2^·s^−1^). Expression levels of *CBDAS* in hemp plants grown under three different light intensities for 35 days during flowering stage. Data were analyzed using one-way ANOVA followed by Tukey’s test. Different letters indicate significant differences between treatments (*P* < 0.01). Data are presented as mean ± standard error (*n* = 3).

We analyzed the expression levels of *GPPS*, a downstream enzyme in the MEP pathway, and *OLS* and *OAC*, which are downstream enzymes in the hexanoate pathway. Additionally, we analyzed *PT*, the enzyme responsible for CBGA biosynthesis, and the major cannabinoid synthases *THCAS*, *CBDAS*, and *CBCAS* in the cannabinoid biosynthesis pathway (**Figure 7A**). Expression analysis revealed that *GPPS* showed the highest expression under 400 μmol·m^−2^·s^−1^, while *OLS*, *OAC*, *PT*, *THCAS*, *CBDAS*, and *CBCAS* exhibited higher expression levels under 600 μmol·m^−2^·s^−1^. Two distinct clusters were observed. One represented the MEP pathway and the other included the hexanoate pathway for cannabinoid biosynthesis (**Figure 7B**). Specifically, *CBDAS* expression, responsible for CBDA production, a key compound in our study, was significantly increased under 600 μmol·m^−2^·s^−1^ (**Figure 8**). These findings suggest that high-intensity light stimulates the hexanoate–OA–CBGA–cannabinoid biosynthesis pathway, thereby enhancing cannabinoid accumulation.

## 4 Discussion

Cultivar genetics and light intensity collectively determine the hemp biomass and quality parameters. High biomass production in plants requires adequate photosynthetic carbon fixation, for which light is a key factor (Mills, 2012). When the light intensity is either too low or too high, photosynthesis is inhibited, leading to reduced plant growth and flowering (Barber and Andersson, 1992). When plants absorb light, carbon dioxide and water are converted to glucose and oxygen through photosynthesis, and glucose is subsequently utilized for plant growth (Gertlowski and Petersen, 1993). These sugars serve as fundamental building blocks for the synthesis of primary and secondary metabolites in plants (Stracke et al., 2007).

In our study, stem, leaf, and flower dry mass showed linear increases as light intensity increased from 200 to 600 μmol·m^−2^·s^−1^. These results are consistent with those of previous studies, which have demonstrated that increased light intensity promotes hemp growth through enhanced photosynthetic capacity. Vanhove et al. (2011) reported that the growth parameters of high-THC cannabis increased linearly with light intensity, with yield per square meter approaching saturation at 600 μmol·m^−2^·s^−1^. Other studies on drug-type cannabis have extended these findings to higher light intensities. Rodriguez-Morrison et al. (2021) demonstrated linear yield increases across 120–1,800 μmol·m^−2^·s^−1^, achieving 450% flower yield (g·m^−2^) increases with no light saturation observed, while Llewellyn et al. (2022) found 60% flower dry mass (g/plant) increases when light intensity increased from 600 to 1,000 μmol·m^−2^·s^−1^ in the high-THC cultivar ‘Meridian’. Additionally, Sae-Tang et al. (2024) demonstrated that medicinal cannabis can efficiently utilize even higher intensities up to 1000 μmol·m^−2^·s^−1^ without saturation. The observed wider internodal spacing under 200 μmol·m^−2^·s^−1^ treatment represents a typical shade avoidance response, where plants elongate internodes under low light conditions to optimize light capture (Ghorbanzadeh et al., 2021; Xu et al., 2021).

Higher light intensities simultaneously increased biomass accumulation and cannabinoid production. Our results demonstrate that CBDA, CBD, and THCA exhibited linear accumulation across the 200–600 μmol·m^−2^·s^−1^ range, with the highest accumulation occurring at 600 μmol·m^−2^·s^−1^. The 36.88% increase in total CBD content at 600 μmol·m^−2^·s^−1^ compared to 200 μmol·m^−2^·s^−1^ demonstrates the positive relationship between light intensity and phytochemical production. This aligns with previous studies on various cannabis types, which show that substantial cannabinoid yields increase at higher light intensities (Rodriguez-Morrison et al., 2021; Llewellyn et al., 2022; Sae-Tang et al., 2024). More importantly, CBD yield per plant showed linear increases across the entire 200–600 μmol·m^−2^·s^−1^ light intensity range (*P_lin_* < 0.001). Given that previous studies have demonstrated continued linear increases at much higher intensities (up to 1,800 μmol·m^−2^·s^−1^), further investigation beyond 600 μmol·m^−2^·s^−1^ is warranted to determine the upper limits of light intensity optimization for CBD production in hemp cultivation. This combined effect of enhanced cannabinoid concentration and increased flower biomass production indicates that light intensity optimization effectively maximizes the harvestable CBD production per plant, providing significant implications for commercial hemp cultivation in controlled-environment systems.

Plant phytochemicals are closely linked to the primary photosynthetic pathways. The content of cannabinoids, a carbon-based phytochemical, is highly dependent on robust plant growth and photosynthetic efficiency. Glucose produced through photosynthesis enters the glycolytic pathway, generating precursors for phytochemical synthesis (Aharoni and Galili, 2011). This process leads to the formation of acetyl-CoA, which plays a key role in OA biosynthesis via the hexanoate pathway (Flores-Sanchez and Verpoorte, 2008). Recent molecular studies have provided quantitative evidence of these biochemical connections. The critical role of hexanoyl-CoA availability has been demonstrated through quantification studies showing levels of 15.5 pmol·g^−1^ fresh weight in hemp flowers, with accumulation patterns directly paralleling CBDA production (Stout et al., 2012). Metabolic engineering has identified specific bottlenecks in OA biosynthesis, achieving an 83-fold increase in production by targeting acetyl-CoA carboxylase, pyruvate dehydrogenase bypass systems, and NADPH-generating malic enzymes (Luo et al., 2022). Although metabolomic research has shown enhanced secondary metabolite production at moderate light intensities for certain compounds, our study demonstrated that cannabinoid-specific optimization requires higher intensities.

Our gene expression analysis revealed an increased expression of cannabinoid biosynthesis genes under high light intensity, providing molecular evidence for enhanced cannabinoid production. The expression patterns revealed two distinct clusters: the MEP pathway (*GPPS*) and the hexanoate–cannabinoid biosynthesis pathway, which included *OLS*, *OAC*, *PT*, *THCAS*, *CBDAS*, and *CBCAS*. While *GPPS* showed the highest expression at 400 μmol·m^−2^·s^−1^, reflecting optimal primary energy production from light, genes involved in cannabinoid biosynthesis were maximally expressed at 600 μmol·m^−2^·s^−1^. In particular, *CBDAS*, the primary gene responsible for CBDA synthesis, showed significant upregulation under 600 μmol·m^−2^·s^−1^, directly correlating with the observed increase in CBDA and total CBD content. This coordinated upregulation of the hexanoate–OA–CBGA–cannabinoid biosynthesis pathway under high light intensity explains the enhanced accumulation of cannabinoids and demonstrates that light intensity is a key factor in hemp phytochemical optimization. These findings align with recent reports in molecular studies that UV radiation can upregulate both *CBDAS* and *THCAS* expression by 4-fold, while enhancing *OAC* and *OLS* expression in cannabis cell cultures (Mansouri et al., 2024). Furthermore, comprehensive RNA-seq analysis has revealed that early enzymatic steps, particularly CBGA production, appear to be more rate-limiting than terminal synthase activities, such as *THCAS*, providing new insights into cannabinoid biosynthesis bottlenecks (Apicella et al., 2022). However, the preferential CBDA accumulation despite concurrent *CBDAS* and *THCAS* upregulation under high light intensity can be attributed to cultivar-specific genotypic and enzymatic characteristics. Hemp cultivar ‘Queen Dream’ can possess functional *CBDAS* alleles while harboring hypoactive *THCAS* variants (Singh et al., 2021), which determines the CBD-dominant chemotype regardless of transcript expression levels (Grassa et al., 2021; Ren et al., 2021). The enhanced CBGA precursor availability under 600 μmol·m^−2^·s^−1^ is preferentially directed toward CBDA synthesis through superior CBDAS enzymatic efficiency in such cultivars, demonstrating that cannabinoid profile determination involves both transcriptional and post-transcriptional regulatory mechanisms.

The molecular mechanisms underlying light-regulated cannabinoid production involve complex spectral-dependent responses. Recent spectral studies have demonstrated that LED treatments rich in blue and UV-A wavelengths can produce 26–38% higher THC content than traditional HPS lighting at equivalent intensities (Islam et al., 2021). These spectrum-dependent responses suggest that optimizing both light intensity and spectral quality could further enhance cannabinoid production beyond the intensity optimization demonstrated in our study. Future research should also consider spectral optimization, as dual red peak spectra (640 + 660 nm) have shown superior performance compared with single-peak configurations (Holweg et al., 2024).

This study focused on the ‘Queen Dream’ cultivar. Future research should validate these light intensity management findings across diverse hemp genotypes and chemotypes. In addition, investigating the interactions between light intensity and spectral quality could refine cultivation protocols to maximize cannabinoid production in controlled environmental systems.

## 5 Conclusion

This study demonstrated that hemp ‘Queen Dream’ showed significant increases in shoot biomass accumulation with increasing light intensity from 200 to 600 μmol·m^−2^·s^−1^. Cannabinoid analysis revealed that CBDA, CBD, and THCA levels increased linearly across this light intensity range, with total CBD showing a 36.88% increase at 600 μmol·m^−2^·s^−1^ compared with that at 200 μmol·m^−2^·s^−1^. CBD yield also increased linearly across the 200-600 μmol·m^−2^·s^−1^ range. Gene expression analysis provided molecular evidence for enhanced cannabinoid production, showing coordinated upregulation of the hexanoate–OA–CBGA–cannabinoid biosynthesis pathway under high light intensity, particularly *CBDAS* expression. These findings demonstrate that optimizing light intensity to 600 μmol·m^−2^·s^−1^ effectively enhances both biomass and cannabinoid accumulation during the flowering stage within the tested range. This study provides valuable insights for establishing controlled-environment agriculture systems to optimize hemp phytochemical production through precise light management. For commercial hemp production targeting high CBD content, maintaining light intensity at 600 μmol·m^−2^·s^−1^ during the 35-day flowering stage showed optimal results within the conditions tested.

## Data Availability

The data presented in the study are deposited in the Figshare repository, accession number https://doi.org/10.6084/m9.figshare.30334864.

## Supplementary material

Supplementary Table 1 Primers used for quantitative reverse-transcription PCR analysis. *THCAS* (tetrahydrocannabinolic acid synthase); *CBCAS* (cannabichromenic acid synthase); *CBDAS* (cannabidiolic-acid synthase); *GPPS* (geranyl pyrophosphate synthase); *OLS* (olivetol synthase); *OAC* (olivetolic acid cyclase); *PT* (geranyl pyrophosphate-olivetolate geranyl transferase)

Supplementary Figure 1 Representative HPLC chromatogram showing separation and identification of cannabinoids in hemp (*Cannabis sativa* ‘Queen Dream’) flower extracts. The chromatogram displays three main cannabinoid peaks: cannabidiol (CBD) at retention time 7.890 min, cannabidiolic acid (CBDA) at 9.401 min, and tetrahydrocannabinolic acid (THCA) at 13.107 min.

## References

[B1] Aharoni A. Galili G. (2011). Metabolic engineering of the plant primary–secondary metabolism interface. Curr. Opin. Biotechnol. 22, 239–244. doi: 10.1016/j.copbio.2010.11.004, PMID: 21144730

[B2] Ahmed S. A. Ross S. A. Slade D. Radwan M. M. Khan I. A. ElSohly M. A. (2015). Minor oxygenated cannabinoids from high potency *Cannabis sativa* L. Phytochemistry 117, 194–199. doi: 10.1016/j.phytochem.2015.04.007, PMID: 26093324 PMC4883105

[B3] Apicella P. J. Skirpan A. L. McClelland A. Xiong Y. Makris T. M. Page J. E. (2022). Delineating genetic regulation of cannabinoid biosynthesis during female flower development in *Cannabis sativa* . Plant Direct 6, e412. doi: 10.1002/pld3.412, PMID: 35774623 PMC9219008

[B4] Aso E. Ferrer I. (2016). CB2 cannabinoid receptor as potential target against Alzheimer’s disease. Front. Neurosci. 10. doi: 10.3389/fnins.2016.00243, PMID: 27303261 PMC4885828

[B5] Barber J. Andersson B. (1992). Too much of a good thing: light can be bad for photosynthesis. Trends Biochem. Sci. 17, 61–66. doi: 10.1016/0968-0004(92)90503-2, PMID: 1566330

[B6] Cho A. R. Song S. J. Chung S. W. Kim Y. J. (2019). CO_2_ enrichment with higher light level improves flowering quality of Phalaenopsis Queen Beer ‘Mantefon’. Sci. Hortic. 247, 356–361. doi: 10.1016/j.scienta.2018.12.030

[B7] Chowdhury M. Kiraga S. Islam M. N. Ali M. Reza M. N. Lee W. H.. (2021). Effects of temperature, relative humidity, and carbon dioxide concentration on growth and glucosinolate content of kale grown in a plant factory. Foods 10, 1524. doi: 10.3390/foods10071524, PMID: 34359392 PMC8306225

[B8] Colonna E. Rouphael Y. Barbieri G. De Pascale S. (2016). Nutritional quality of ten leafy vegetabl es harvested at two light intensities. Food Chem. 199, 702–710. doi: 10.1016/j.foodchem.2015.12.068, PMID: 26776027

[B9] Crispim Massuela D. Hartung J. Munz S. Erpenbach F. Graeff-Hönninger S. (2022). Impact of harvest time and pruning technique on total CBD concentration and yield of medicinal cannabis. Plants 11, 140. doi: 10.3390/plants11010140, PMID: 35009146 PMC8747189

[B10] Eichhorn Bilodeau S. Wu B. S. Rufyikiri A. S. MacPherson S. Lefsrud M. (2019). An update on plant photobiology and implications for cannabis production. Front. Plant Sci. 10. doi: 10.3389/fpls.2019.00296, PMID: 31001288 PMC6455078

[B11] ElSohly M. A. Radwan M. M. Gul W. Chandra S. Galal A. (2017). “Phytochemistry of *Cannabis sativa* L,” in Phytocannabinoids: Unraveling the Complex Chemistry and Pharmacology of Cannabis sativa. (Cham, Switzerland: Springer), 1–36. 10.1007/978-3-319-45541-9_1 28120229

[B12] Fellermeier M. Eisenreich W. Bacher A. Zenk M. H. (2001). Biosynthesis of cannabinoids: incorporation experiments with ¹³C-labeled glucoses. Eur. J. Biochem. 268, 1596–1604. doi: 10.1046/j.1432-1327.2001.02046.x, PMID: 11248677

[B13] Flores-Sanchez I. J. Verpoorte R. (2008). Secondary metabolism in cannabis. Phytochem. Rev. 7, 615–639. doi: 10.1007/s11101-008-9094-4

[B14] Gertlowski C. Petersen M. (1993). Influence of the carbon source on growth and rosmarinic acid production in suspension cultures of *Coleus blumei* . Plant Cell Tissue Organ Cult. 34, 183–190. doi: 10.1007/BF00036100

[B15] Ghorbanzadeh P. Aliniaeifard S. Esmaeili M. Mashal M. Azadegan B. Seif M. (2021). Dependency of growth, water use efficiency, chlorophyll fluorescence, and stomatal characteristics of lettuce plants to light intensity. J. Plant Growth Regul. 40, 2191–2207. doi: 10.1007/s00344-020-10269-z

[B16] Grassa C. J. Weiblen G. D. Wenger J. P. Dabney C. Poplawski S. G. Motley S. T.. (2021). A new Cannabis genome assembly associates elevated cannabidiol (CBD) with hemp introgressed into marijuana. New Phytol. 230, 1665–1679. doi: 10.1111/nph.17243, PMID: 33521943 PMC8248131

[B17] Hacke A. C. M. Lima D. de Costa F. Deshmukh K. Li N. Chow A. M.. (2019). Probing the antioxidant activity of Δ9-tetrahydrocannabinol and cannabidiol in *Cannabis sativa* extracts. Analyst 144, 4952–4961. doi: 10.1039/C9AN00890J, PMID: 31318364

[B18] Hahm S. Lee J. Y. Im H. M. Lee H. J. Park J. (2025). Influence of temperature stress on the major cannabinoid contents and biosynthesis gene expression levels in industrial hemp (*Cannabis sativa* L.). Hortic. Sci. Technol. 43, 221–233. doi: 10.7235/HORT.20250024

[B19] Holweg M. M. S. F. Kaiser E. Kappers I. F. Heuvelink E. Marcelis L. F. M. (2024). The role of red and white light in optimizing growth and accumulation of plant specialized metabolites at two light intensities in medical cannabis (*Cannabis sativa* L.). Front. Plant Sci. 15. doi: 10.3389/fpls.2024.1393803, PMID: 38957608 PMC11217568

[B20] Islam N. Amin M. N. Siddiquee M. A. Pearson S. Garman P. Kristiansen B.. (2021). LED lights to maximize the cannabinoid production in Cannabis. Biology 10, 710. doi: 10.3390/biology10080710, PMID: 34439943 PMC8389281

[B21] Kolodinsky J. Lacasse H. (2021). Consumer response to hemp: A case study of Vermont residents from 2019 to 2020. Glob. Change Biol. Bioenergy 13, 537–545. doi: 10.1111/gcbb.12786

[B22] Kow R. L. Jiang K. Naydenov A. V. Le J. H. Stella N. Nathanson N. M. (2014). Modulation of pilocarpine-induced seizures by cannabinoid receptor 1. PloS One 9, e95922. doi: 10.1371/journal.pone.0095922, PMID: 24752144 PMC3994118

[B23] Kozai T. (2013). Resource use efficiency of closed plant production system with artificial light: Concept, estimation and application to plant factory. Proc. Jpn. Acad. 89, 447–461. doi: 10.2183/pjab.89.447, PMID: 24334509 PMC3881955

[B24] Llewellyn D. Golem S. Foley E. Dinka S. Jones A. M. P. Zheng Y. (2022). Indoor grown cannabis yield increased proportionally with light intensity, but ultraviolet radiation did not affect yield or cannabinoid content. Front. Plant Sci. 13. doi: 10.3389/fpls.2022.974018, PMID: 36237501 PMC9551646

[B25] Luo X. Reiter M. A. d’Espaux L. Wong J. Denby C. M. Lechner A. (2019). Complete biosynthesis of cannabinoids and their unnatural analogues in yeast. Nature 567, 123–126. doi: 10.1038/s41586-019-0978-9, PMID: 30814733

[B26] Luo X. Reiter M. A. d’Espaux L. Wong J. Denby C. M. Lechner A.. (2022). Biosynthesis of cannabinoid precursor olivetolic acid in genetically engineered *Yarrowia lipolytica* . Commun. Biol. 5, 1262. doi: 10.1038/s42003-022-04202-1, PMID: 36371560 PMC9653464

[B27] Mansouri H. Asrar Z. Amarowicz R. (2024). Low UV radiation influenced DNA methylation, gene regulation, cell proliferation, viability, and biochemical differentiation in the cell suspension cultures of *Cannabis indica.* J. Photochem. Photobiol. B 250, 112829. doi: 10.1016/j.jphotobiol.2024.112829, PMID: 38569457

[B28] Mawphlang O. I. Kharshiing E. V. (2017). Photoreceptor mediated plant growth responses: implications for photoreceptor engineering toward improved performance in crops. Front. Plant Sci. 8. doi: 10.3389/fpls.2017.01181, PMID: 28744290 PMC5504655

[B29] Mills E. (2012). The carbon footprint of indoor Cannabis production. Energy Policy 46, 58–67. doi: 10.1016/j.enpol.2012.03.023

[B30] Mostafavi M. Gaitanis J. (2020). Autism spectrum disorder and medical cannabis: review and clinical experience. Semin. Pediatr. Neurol. 35, 100833. doi: 10.1016/j.spen.2020.100833, PMID: 32892960

[B31] Nelson D. C. Flematti G. R. Riseborough J. A. Ghisalberti E. L. Dixon K. W. Smith S. M. (2010). Karrikins enhance light responses during germination and seedling development in *Arabidopsis thaliana* . Proc. Natl. Acad. Sci. 107, 7095–7100. doi: 10.1073/pnas.0911635107, PMID: 20351290 PMC2872431

[B32] Nelson K. M. Bisson J. Singh G. Graham J. G. Chen S. N. Friesen J. B.. (2020). The essential medicinal chemistry of cannabidiol (CBD). J. Med. Chem. 63, 12137–12155. doi: 10.1021/acs.jmedchem.0c00724, PMID: 32804502 PMC7666069

[B33] Pérez-López U. Sgherri C. Miranda-Apodaca J. Micaelli F. Lacuesta M. Mena-Petite A.. (2018). Concentration of phenolic compounds is increased in lettuce grown under high light intensity and elevated CO_2_ . Plant Physiol. Biochem. 123, 233–241. doi: 10.1016/j.plaphy.2017.12.010, PMID: 29253801

[B34] Pisanti S. Malfitano A. M. Ciaglia E. Lamberti A. Ranieri R. Cuomo G.. (2017). Cannabidiol: State of the art and new challenges for therapeutic applications. Pharmacol. Ther. 175, 133–150. doi: 10.1016/j.pharmthera.2017.02.041, PMID: 28232276

[B35] Ren G. Zhang X. Li Y. Ridout K. Serrano-Serrano M. L. Yang Y.. (2021). Large-scale whole-genome resequencing unravels the domestication history of *Cannabis sativa* . Sci. Adv. 7, eabg2286. doi: 10.1126/sciadv.abg2286, PMID: 34272249 PMC8284894

[B36] Rodriguez-Morrison V. Llewellyn D. Zheng Y. (2021). Cannabis yield, potency, and leaf photosynthesis respond differently to increasing light levels in an indoor environment. Front. Plant Sci. 12. doi: 10.3389/fpls.2021.646020, PMID: 34046049 PMC8144505

[B37] Sabatino L. Ntatsi G. Iapichino G. D’Anna F. De Pasquale C. (2019). Effect of selenium enrichment and type of application on yield, functional quality and mineral composition of curly endive grown in a hydroponic system. Agronomy 9, 207. doi: 10.3390/agronomy9040207

[B38] Sae-Tang W. Heuvelink E. Nicole C. C. S. Kaiser E. Sneeuw K. Holweg M. M. S. F.. (2024). High light intensity improves yield of specialized metabolites in medicinal cannabis (*Cannabis sativa* L.), resulting from both higher inflorescence mass and concentrations of metabolites. J. Appl. Res. Med. Aromat. Plants 43, 100583. doi: 10.1016/j.jarmap.2024.100583

[B39] Singh A. Bilichak A. Kovalchuk I. (2021). The genetics of Cannabis—genomic variations of key synthases and their effect on cannabinoid content. Genome 64, 490–501. doi: 10.1139/gen-2020-0087, PMID: 33186070

[B40] Small E. (1976). Cronquist, A. A practical and natural taxonomy for Cannabis. Taxon 25, 405–435. doi: 10.2307/1220524

[B41] Stout J. M. Boubakir Z. Ambrose S. J. Purves R. W. Page J. E. (2012). The hexanoyl-CoA precursor for cannabinoid biosynthesis is formed by an acyl-activating enzyme in *Cannabis sativa* trichomes. Plant J. 71, 353–365. doi: 10.1111/j.1365-313X.2012.04949.x, PMID: 22353623

[B42] Stracke R. Ishihara H. Huep G. Barsch A. Mehrtens F. Niehaus K.. (2007). Differential regulation of closely related R2R3-MYB transcription factors controls flavonol accumulation in different parts of the *Arabidopsis thaliana* seedling. Plant J. 50, 660–677. doi: 10.1111/j.1365-313X.2007.03078.x, PMID: 17419845 PMC1976380

[B43] Tagen M. Klumpers L. E. (2022). Review of delta-8-tetrahydrocannabinol (Δ8-THC): Comparative pharmacology with Δ9-THC. Br. J. Pharmacol. 179, 3915–3933. doi: 10.1111/bph.15865, PMID: 35523678

[B44] Tahir M. N. Shahbazi F. Rondeau-Gagné S. Trant J. F. (2021). The biosynthesis of the cannabinoids. J. Cannabis Res. 3, 7. doi: 10.1186/s42238-021-00062-4, PMID: 33722296 PMC7962319

[B45] Taura F. Tanaka S. Taguchi C. Fukamizu T. Tanaka H. Shoyama Y.. (2009). Characterization of olivetol synthase, a polyketide synthase putatively involved in cannabinoid biosynthetic pathway. FEBS Lett. 583, 2061–2066. doi: 10.1016/j.febslet.2009.05.024, PMID: 19454282

[B46] Teleszko M. Zając A. Rusak T. (2022). Hemp seeds of the polish ‘Bialobrzeskie’ and ‘Henola’ varieties (*Cannabis sativa* L. var. sativa) as prospective plant sources for food production. Molecules 27, 1448. doi: 10.3390/molecules27041448, PMID: 35209234 PMC8880225

[B47] Tian Z. Ma W. Yang Q. Duan F. (2021). Application status and challenges of machine vision in plant factory—A review. Inf. Process. Agric. 9, 195–211. doi: 10.1016/j.inpa.2021.06.003

[B48] Tombesi S. Palliotti A. Poni S. Farinelli D. (2015). Influence of light and shoot development stage on leaf photosynthesis and carbohydrate status during the adventitious root formation in cuttings of *Corylus avellana* L. Front. Plant Sci. 6. doi: 10.3389/fpls.2015.00973, PMID: 26635821 PMC4654426

[B49] UNODC (2009). World drug report (Vienna, Austria: United Nations, United Nations).

[B50] Vanhove W. Van Damme P. Meert N. (2011). Factors determining yield and quality of illicit indoor cannabis (Cannabis spp.) production. Forensic Sci. Int. 212, 158–163. doi: 10.1016/j.forsciint.2011.06.006, PMID: 21737218

[B51] Vigil J. M. Stith S. S. Diviant J. P. Brockelman F. Keeling K. Hall B. (2018). Effectiveness of raw, natural medical cannabis flower for treating insomnia under naturalistic conditions. Medicines 5, 75. doi: 10.3390/medicines5030075, PMID: 29997343 PMC6164964

[B52] Wilkinson J. D. Williamson E. M. (2007). Cannabinoids inhibit human keratinocyte proliferation through a non-CB1/CB2 mechanism and have a potential therapeutic value in the treatment of psoriasis. J. Dermatol. Sci. 45, 87–92. doi: 10.1016/j.jdermsci.2006.10.009, PMID: 17157480

[B53] Xu Y. Wang C. Zhang R. Ma C. Dong S. Gong Z. (2021). The relationship between internode elongation of soybean stems and spectral distribution of light in the canopy under different plant densities. Plant Prod. Sci. 24, 326–338. doi: 10.1080/1343943X.2020.1847666

[B54] Zhang X. He D. Niu G. Yan Z. Song J. (2018). Effects of environment lighting on the growth, photosynthesis, and quality of hydroponic lettuce in a plant factory. Int. J. Agric. Biol. Eng. 11, 33–40. doi: 10.25165/j.ijabe.20181102.3420

