# Supplementary material for: High light intensity enhances cannabinoid biosynthesis through concerted gene expression in hemp (Cannabis sativa) flowers
Source: Front Plant Sci. 2025 Oct 21;16:1687794. doi: 10.3389/fpls.2025.1687794 (PMC12583074; doi:10.3389/fpls.2025.1687794)
Supplement: Supplementary Table 1 — Primers used for quantitative reverse-transcription PCR analysis. THCAS (tetrahydrocannabinolic acid synthase); CBCAS (cannabichromenic acid synthase); CBDAS (cannabidiolic-acid synthase); GPPS (geranyl pyrophosphate synthase); OLS (olivetol synthase); OAC (olivetolic acid cyclase); PT (geranyl pyrophosphate-olivetolate geranyl transferase) [file DataSheet1.docx]

**Supplementary Table 1.** Primers used for quantitative reverse-transcription PCR analysis. *THCAS* (tetrahydrocannabinolic acid synthase); *CBCAS* (cannabichromenic acid synthase); *CBDAS* (cannabidiolic-acid synthase); *GPP* (geranyl pyrophosphate) synthase; *OLS* (olivetol synthase); *OAC* (olivetolic acid cyclase); *PT* (geranyl pyrophosphate-olivetolate geranyl transferase)

| **Gene** | **Primer Sequence (5´ to 3´)** | |
| --- | --- | --- |
|  | **Forward Primer** | **Reverse Primer** |
| ***GPPS*** | GTGAACTCGTAGGCGGAAAA | ATACAAGGGAGGTCGTCGTG |
| ***OLS*** | AGTCCCTCAGTGAAGCGTGT | GCGCCTTTGTTATTCTCTGC |
| ***OAC*** | CACAGAAGCCCAAAAGGAAG | TCTTTCATGGCTGGGATGAT |
| ***PT*** | CTGAGCCTCCAGAATCTGATAATC | GCCTTGAACATCAGAGACCAAC |
| ***THCAS*** | CGAAAATCCATGGGAGAAGA | ACTTTGATGGGACAGCAACC |
| ***CBCAS*** | AGGAGGCTATGGAGCATTGA | TCTTCTCCCATGGATTTTCG |
| ***CBDAS*** | GAGGCTATGGACCATTGA | GGACAGCAACCAGTCTAA |


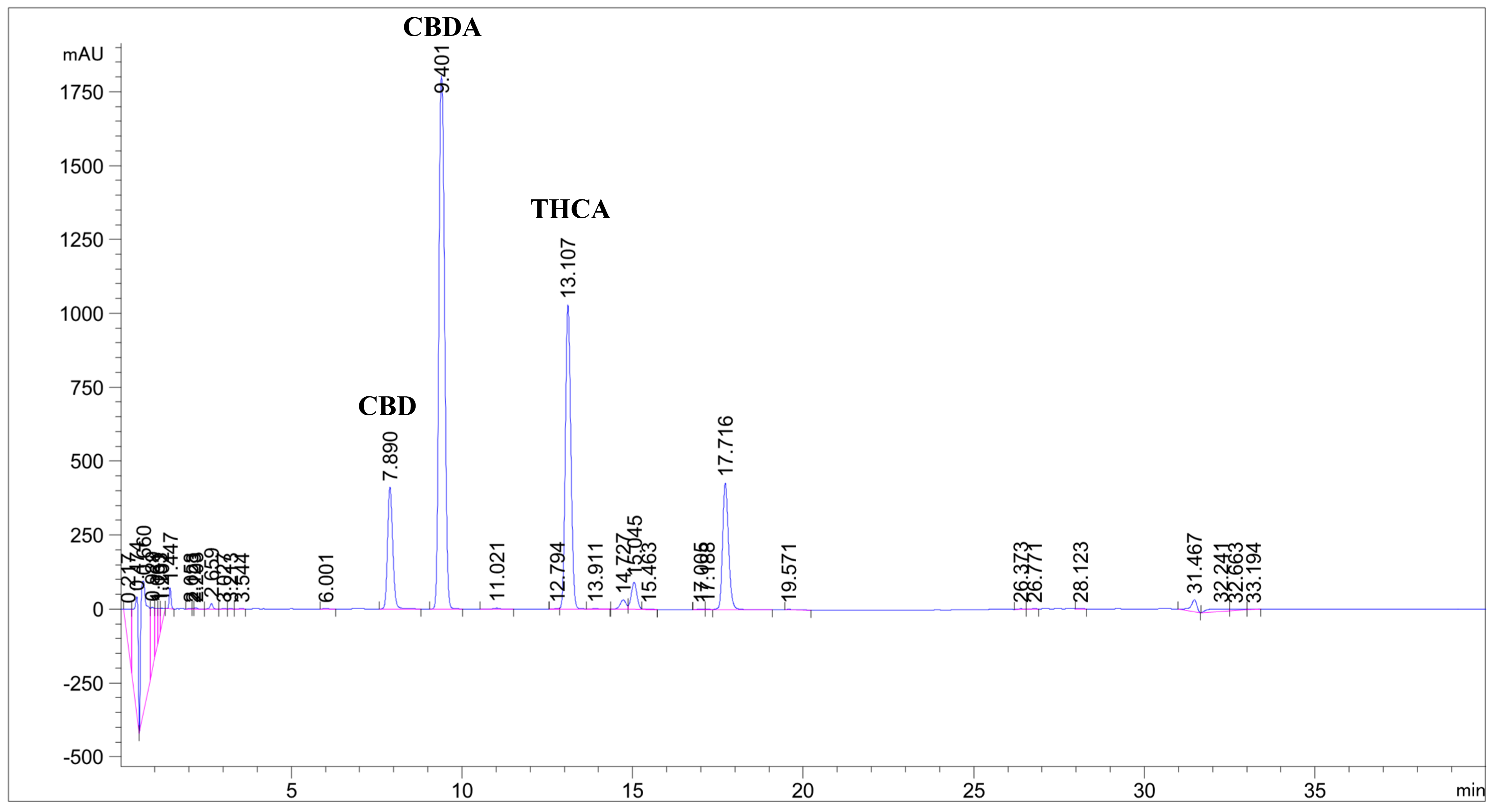


**Supplementary Figure 1.** Representative HPLC chromatogram showing separation and identification of cannabinoids in hemp (*Cannabis sativa* 'Queen Dream') flower extracts. The chromatogram displays three main cannabinoid peaks: cannabidiol (CBD) at retention time 7.890 min, cannabidiolic acid (CBDA) at 9.401 min, and tetrahydrocannabinolic acid (THCA) at 13.107 min.
